# Mechanism of Zhinao Capsule in Treating Alzheimer's Disease Based on Network Pharmacology Analysis and Molecular Docking Validation

**DOI:** 10.1155/2022/5708769

**Published:** 2022-08-18

**Authors:** Yanzhen Ma, Shaopeng Huang, Hui Jiang, Wenming Yang

**Affiliations:** ^1^Experimental Center of Clinical Research, The First Affiliated Hospital of Anhui University of Chinese Medicine, Hefei, Anhui 230031, China; ^2^College of Pharmacy, The Anhui University of Chinese Medicine, Hefei 230031, Anhui, China; ^3^Key Laboratory of Xin'an Ministry of Medical Education, The First Affiliated Hospital of Anhui University of Chinese Medicine, Hefei, Anhui 230031, China

## Abstract

**Objective:**

This study aimed to determine the active components of Zhinao capsule (ZNC) and the targets in treating Alzheimer's disease (AD) so as to investigate and explore the mechanism of ZNC for AD.

**Methods:**

The active components and targets of ZNC were determined from the traditional Chinese medicine systems pharmacology database (TCMSP). The target genes of AD were searched for in GeneCards. Cytoscape was used to construct an herb-component-target-disease network. A protein-protein interaction (PPI) network was constructed by STRING. Gene ontology (GO) and Kyoto Encyclopedia of Genes and Genomes (KEGG) pathway enrichment analyses were performed using the OmicShare. UCSF Chimera and SwissDock were used for molecular docking verification. Finally, four key target genes were validated by Western blotting.

**Results:**

In total, 55 active components, 287 targets of active components, 1197 disease genes, and 134 common genes were screened, which were significantly enriched in 3975 terms of biological processes (BP), 284 terms of cellular components (CC), 433 terms of molecular functions (MF), and 245 signaling pathways. Caspase-3 (CASP3) and beta-sitosterol, tumor necrosis factor-alpha (TNF-*α*) and quercetin, vascular endothelial growth factor A (VEGFA) and baicalein, and mitogen-activated protein kinase 1 (MAPK1) and quercetin showed good-to-better docking. Moreover, ZNC not only downregulated CASP3 and TNF-*α* protein expression but also upregulated the protein expression of VEGFA and MAPK1.

**Conclusions:**

The active components of ZNC, such as beta-sitosterol, quercetin, and baicalein may act on multiple targets like CASP3, VEGFA, MAPK1, and TNF-*α* to affect T cell receptor (TCR), TNF, and MAPK signaling pathway, thereby achieving the treatment of AD. This study provides a scientific basis for further exploring the potential mechanism of ZNC in the treatment of AD and a reference for its clinical application.

## 1. Introduction

Alzheimer's disease (AD) is one of the most common neurodegenerative diseases, which is clinically characterized by a progressive decline in cognitive function [1]. The histopathological hallmarks of AD are neuritic plaque and neurofibrillary tangle (NFT) formation in the brain [2]. The cost of treatment continues to increase and results in a significant burden on both families and society [3].

Currently, one effective treatment for AD is drug therapy, which can slightly alleviate symptoms and prevent or slow AD progression. Traditional Chinese medicine (TCM) formulas have long been used to prevent and treat a variety of diseases, including AD [4–6]. Unlike other drugs, TCM formulas have multiple targets, low toxicity, and strong regulatory effects. Therefore, they have attracted increasing attention [7, 8].

Zhinao capsule (ZNC), a Chinese patent medicine, is mainly composed of Dangshen (DS, *Codonopsis pilosula*), Huangqi (HQ, *Astragali membranaceus*), Huangjing (HJ, *Polygonatum sibiricum*), Rou Congrong (RCR, *Cistanche deserticola*), Yujin (YJ, *Curcuma wenyujin*), Shi Changpu (SCP, *Acorus tatarinowii*), Chuanxiong (CX, *Ligusticum chuanxiong*), and Dilong (DL, *Pheretima aspergillum*) and has good efficacy in treating AD [9]. Owing to the complexity of Chinese patent medicines, it is challenging to identify the mechanism of ZNC through conventional pharmacological methods because of its multiple components, multiple targets, and multiple pathways.

Network pharmacology is a systematic analytical method based on systems biology that combines polypharmacology, molecular network data, bioinformatics, and computer simulations [10]. And it is a new method based on the multidisciplinary fusion theory of “active ingredient-target-disease” and interaction network [11]. In recent years, network pharmacology has become a powerful tool that can be combined with pharmacology [12]. This method has been shown to be an efficient way to explore the multiple components, targets, and pathways of TCM formulas [13]. For example, Cao et al. [14] applied network pharmacology and demonstrated that the active components of Yinqiaosan, that is, luteolin, naringenin, and farnesin, can act on multiple targets, such as tumor necrosis factor (TNF), mitogen-activated protein kinase 1 (MAPK1), and Caspase-3 (CASP3), to regulate signaling pathways, such as Kaposi sarcoma-associated herpesvirus infection, advanced glycation end products (AGE), and their receptors, thereby curing COVID-19. Cui et al. [15] reported that the active components of Huangqi Sijunzi decoction, including quercetin, luteolin, kaempferol, and naringenin, might exert therapeutic effects against cancer-related fatigue by suppressing inflammatory responses and the expression of tumor-related genes such as vascular endothelial growth factor A (VEGFA) and CASP3. Wang et al. [16] found via network pharmacology that the active components of Xiaokui Jiedu decoction, such as *β*-sitosterol and quercetin, may alleviate ulcerative colitis by acting on multiple key targets to regulate oxidative stress-related pathways.

In this study, we hypothesized that ZNC may exert a therapeutic effect against AD development through the ability of its key components to act on key targets and signaling pathways. However, what are active ingredients, critical targets, and signaling pathways of ZNC is still unclear so far. Therefore, in order to explore this question, network pharmacology was used to investigate the potential mechanisms of ZNC treatment of AD, which provides a scientific basis for subsequent experimental research.

## 2. Materials and Methods

### 2.1. Screening of Active Components and Related Targets of ZNC

The traditional Chinese medicine systems pharmacology database (TCMSP) (http://lsp.nwu.edu.cn/tcmsp.php) [17], which is a powerful knowledge repository and analysis platform for Chinese medicines and related compounds, was used to identify the active components in ZNC. The network between herbs and components is shown in the UpSet plot. Based on the literature [18], an oral bioavailability (OB) ≥30% and drug-likeness (DL) ≥ 0.18 were set as the screening criteria. Next, the normalized gene names of the potential target proteins were obtained by searching the UniProt database (https://www.uniprot.org/) [19] for further analysis. The network between herbs and active components was visualized with Cytoscape 3.7.2 [20].

### 2.2. Screening of Disease-Related Targets

GeneCards (https://www.genecards.org/) [21] is an integrative database that provides a comprehensive map of human genes associated with diseases. The target proteins of AD were searched in GeneCards (https://www.genecards.org/) using “Alzheimer's disease” as the keyword.

### 2.3. Filtering of Intersecting Targets and Protein-Protein Interaction (PPI) Network Construction

We input the active components and AD-related targets of ZNC into the Venn tool Venny 2.1.0 (http://bioinformatics.psb.ugent.be/webtools/Venn/) to obtain intersecting targets. The common targets of ZNC and AD were imported into the STRING (https://string-db.org/) [22] platform, which is a database that is used to show protein-protein interactions. Then, we input the data from STRING into Cytoscape to visualize the PPI network to screen key targets according to the degree for further analysis.

### 2.4. Gene Ontology (GO) and Kyoto Encyclopedia of Genes and Genomes (KEGG) Pathway Enrichment Analysis

The targets were entered into the online software OmicShare (https://www.omicshare.com/) [23] to identify the biological functions such as molecular functions (MF), cellular components (CC), and biological processes (BP) terms, and *P* value <0.05 indicated significant enrichment.

### 2.5. Molecular Docking

SwissDock (http://swissdock.ch/) [24] was used to perform molecular docking of the key active components of ZNC and potential therapeutic targets. To carry out docking analysis, the structures of candidate targets were downloaded from the protein data bank (PDB) (https://www.rcsb.org/) [25], and water molecules and organics were removed using PyMOL (http://www.pymol.org/) [26]. The structures of output relative ligands were saved in MOL2 format. After the ligand format and the target proteins format were downloaded, molecular docking was performed. UCSF Chimera software was used to visualize and analyze the docking results. The lowest binding energy (kcal/mol) was used as the standard comparison.

### 2.6. Western Blotting

SPF-grade ICR mice were intraperitoneally injected with D-galactose and sodium nitrite once per day for 6 weeks to establish an AD model [27]. From the day of model induction, the mice were intragastrically administered ZNC (3.90 g/kg) once per day for 6 weeks. The experimental protocol was approved by the Animal Ethics Committee of Anhui University of Chinese Medicine and is shown in Supplementary Material Figure S1.

The hippocampal tissue was lysed in RIPA buffer and centrifuged at 12000 rpm for 10 min to collect the supernatant. The protein samples were boiled for 5–10 min, separated on SDS-polyacrylamide gels, and transferred to a PVDF membrane. The membranes were blocked with 5% skim milk powder for 2 h and then incubated with primary antibodies (*β*-actin, CASP3, TNF-*α*, VEGFA, and MAPK1, 1 : 1000) at 4°C overnight. The membranes were washed with TBST and incubated with the corresponding secondary antibodies for 2 h at room temperature. The proteins were visualized with the ECL reagent, detected by an automatic exposure instrument, and quantitatively analyzed by ImageJ software.

### 2.7. Statistical Analysis

All statistical analyses were performed using SPSS 25.0 software. The measurement data are expressed as $\bar{x}\pm s$. One-way analysis of variance (ANOVA) and Fisher's least significant difference (LSD) were used. *P* value <0.05 was considered statistically significant.

**Figure 1:**
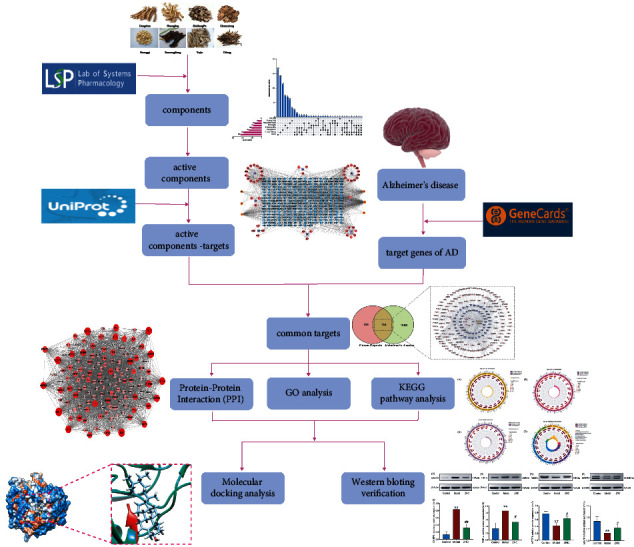
Technical scheme. Network pharmacology analysis of the mechanisms of ZNC in the treatment of AD.

The research process for exploring the potential mechanisms of ZNC treating AD is shown in Figure 1.

## 3. Results

### 3.1. Screening of the Active Components and Targets of ZNC

**Figure 2:**
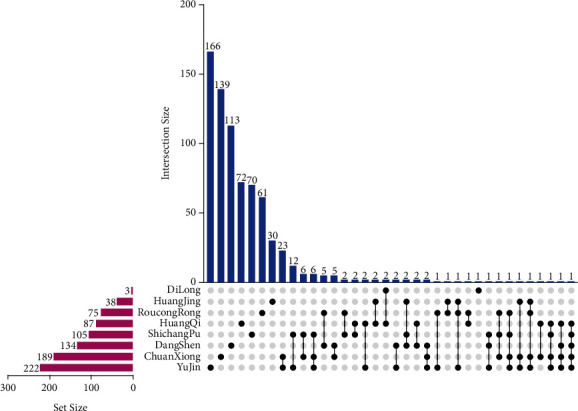
Chart showing the intersection between components and herbs. The common components of different herbs are shown in the top bar chart. The solid point below the bar chart represents the specific name of each herb.

A total of 853 components of ZNC were screened from the TCMSP database. More than 10% of the components were found in two or more herbs, and different combinations of herbs may provide the greatest effect. Moreover, some herbs have unique components. For example, 166 components were found only in Yujin (Figure 2).

**Figure 3:**
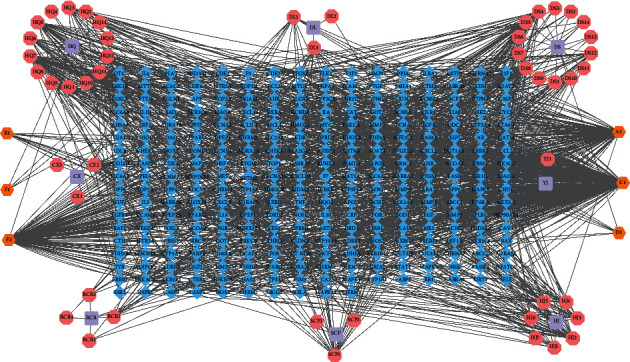
Construction of the ZNC active component-target network. The violet rounded rectangle represents herbs in ZNC, the pink octagon represents active components in those herbs, the azure diamond represents the predicted targets, and orange represents common active components among herbs.

Among the components, there were 55 potential components that met the screening criteria of an OB ≥ 30% and DL ≥ 0.18 (Supplementary Table 1). In addition, 287 targets of the active components were obtained from the TCMSP, which were standardized using the UniProt database. The active components and associated targets were used to construct a component-target network using Cytoscape 3.7.2 (Figure 3).

### 3.2. Collection of Disease Targets and Common Component-Disease Targets

**Figure 4:**
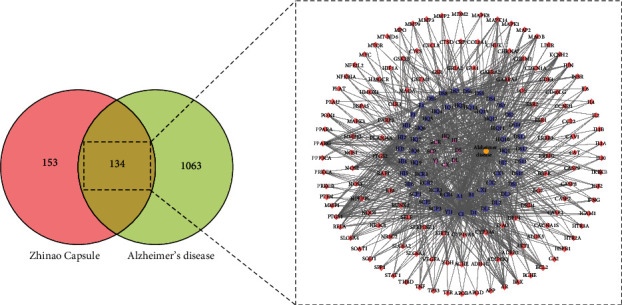
Venn diagram of common component-disease-related targets. The red triangle represents herbs in ZNC, and the blue square represents components of those herbs. The yellow dot represents disease, and the pink rhombus represents common component-disease targets.

A total of 1197 AD-related targets were collected from the GeneCards database. Among them, 134 overlapping targets between ZNC and AD were obtained by the Venn online tool. Subsequently, to determine the mechanism by which ZNC can treat AD, an herb-component-target-disease network was constructed (Figure 4).

### 3.3. PPI Network

**Figure 5:**
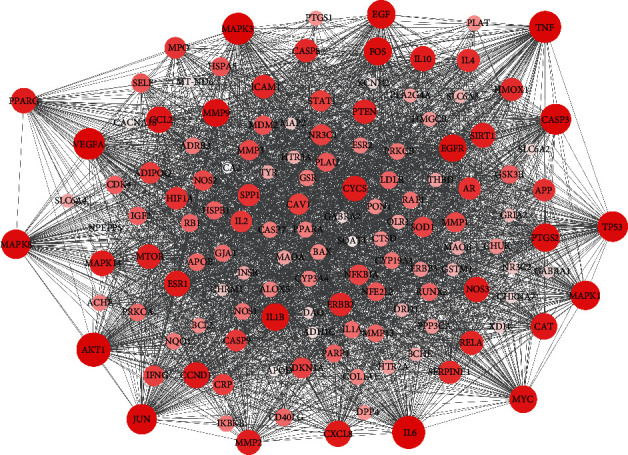
The PPI network. As the degree value increases, the color of the node changes from white to red, and the node size changes from small to large.

To further explore the associations between genes, the common targets were imported into the STRING database to construct a PPI network, which was visualized in Cytoscape (Figure 5). The resulting network included 134 nodes and 2682 edges. According to the screening threshold of an average node degree of 40, 57 targets were identified, which are ranked by degree in Table 1.

### 3.4. GO and KEGG Pathway Enrichment Analysis

**Figure 6:**
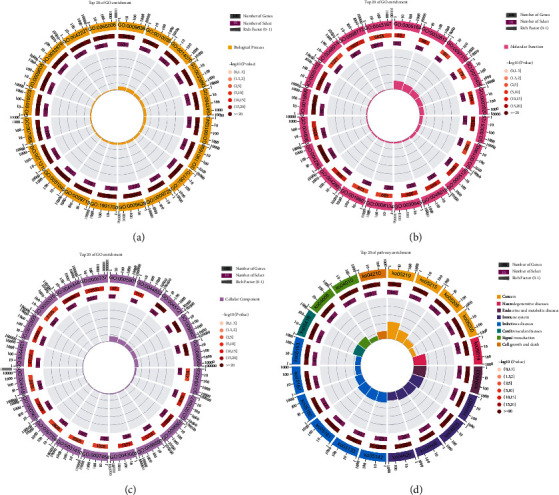
Functional enrichment analysis. (a) The top 20 enriched BPs; (b) the top 20 enriched MFs; (c) the top 20 enriched CCs; (d) the top 20 enriched pathways.

GO and KEGG enrichment analysis was performed using the OmicShare online tool. GO enrichment analysis revealed a total of 5764 enriched BP terms. Among them, 3975 terms were significantly enriched. The top 20 BP terms are shown in Figure 6(a). There was a total of 726 enriched MF terms, including 433 that were significantly enriched. The top 20 MF terms are shown in Figure 6(b). A total of 512 CC terms were enriched, among which 284 were significantly enriched. The top 20 CC terms are shown in Figure 6(c). Simultaneously, KEGG analysis showed that a total of 245 pathways were enriched, including 171 pathways that were significantly enriched. The top 20 pathways are shown in Figure 6(d).

### 3.5. Molecular Docking Analysis

According to the PPI network, 10 targets with higher degrees were used for molecular docking analysis. Based on the SwissDock calculation, those targets and respective components were ranked by binding energy as shown in Supplementary Table 2. The binding energy was obtained by molecular docking to estimate the interaction energy between the active components of ZNC and the potential targets.

**Figure 7:**
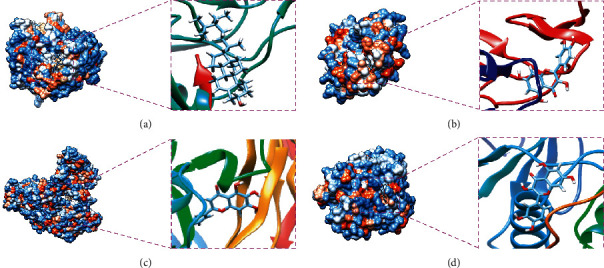
Molecular docking of ZNC components and AD-related targets using the SwissDock server. (a) CASP3 and beta-sitosterol; (b) TNF-*α* and quercetin; (c) VEGFA and baicalein; (d) MAPK1 and quercetin.

Finally, four key targets with a lower binding energy were selected for further analysis. The molecular docking results of these targets (CASP3, TNF-*α*, VEGFA, and MAPK1) are shown in Table 2. In addition, visualizations of the most favorable binding modes of the four key targets are shown in Figure 7.

### 3.6. Effects of ZNC Administration on the Protein Expression of CASP3, TNF-*α*, VEGFA, and MAPK1 in the AD Model Mice

**Figure 8:**
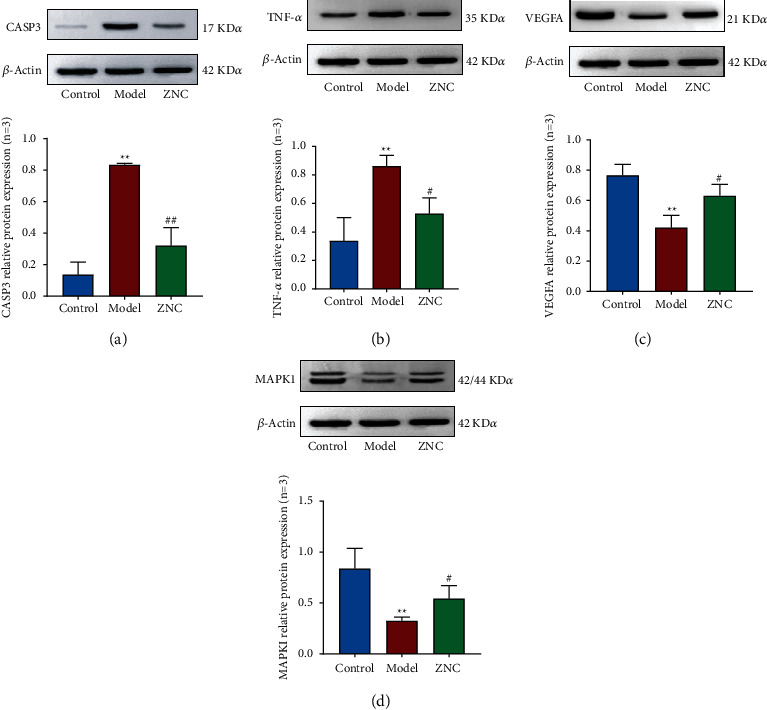
Effects of ZNC on CASP3, TNF-*α*, VEGFA, and MAPK1 protein expression *in vivo*. (a) The protein level of CASP3; (b) the protein level TNF-*α*; (c) the protein level of VEGFA; (d) the protein level of MAPK1. Compared with the control group, ^*∗∗*^*P* < 0.01. Compared with the model group, ^#^*P* < 0.05, ^##^*P* < 0.01.

As shown in Figure 8, the protein expression levels of CASP3 and TNF-*α* in the model group were significantly higher than those in the control group. However, the levels of these proteins in the ZNC group were significantly lower than those in the model group. Compared with those in the control group, the protein expression levels of VEGFA and MAPK1 in the model group were significantly decreased. Compared with those in the model group, the protein expression levels of VEGFA and MAPK1 in the ZNC group were significantly increased.

## 4. Discussion

Network pharmacology is a modern approach for exploring the potential targets of active components [28, 29]. It is well known that TCM formulas have the advantages of multiple components, targets, and pathways which are suitable for the treatment of complex diseases [30, 31]. Network pharmacology is a new approach for studying TCM formulas and identifying the “multicomponent, multitarget” potential of TCMs as therapeutics [32, 33]. In this study, network pharmacology, molecular docking, and animal experiments were used to identify the active components, targets, and signaling pathways of ZNC to explore its potential mechanisms in the treatment of AD.

First, 55 active components of ZNC with an OB ≥ 30% and DL ≥ 0.18 were identified, including beta-sitosterol, baicalein, and quercetin. One study showed that the synergy-based combination of beta-sitosterol successfully enhanced memory and learning by abating free radical and acetylcholine levels in memory loss rats [34]. Another study demonstrated that beta-sitosterol has the potential to treat memory decline similar to that observed in AD [35]. Some studies have indicated that beta-sitosterol alleviates memory and learning impairment in mice by decreasing amyloid-*β* (A*β*) deposition [36]. Baicalein is an oral bioactive agent that may be able to treat AD [37]. Evidence indicates that baicalein notably ameliorates behavioral and cognitive impairment in AD rats [38]. An experimental study has shown that baicalein improves A*β*-induced memory deficits and neuronal atrophy via inhibition of phosphodiesterase 2 (PDE2) and PDE4 [39]. Quercetin protects against the effects of harmful substances and has multiple beneficial effects in AD [40]. Quercetin reverses the histological features of AD and has a protective effect on the cognitive function in older mice [41]. Evidence has shown that quercetin improves cognitive impairment in aging mice by suppression of nod-like receptor protein 3 (NLRP3) inflammasome activation [42]. Therefore, beta-sitosterol, baicalein, and quercetin may be the active components of ZNC in the treatment of AD.

The PPI network contained 134 targets, which represent the most likely core targets of ZNC in the treatment of AD. KEGG pathway enrichment analysis showed that the T cell receptor (TCR), TNF, and the MAPK signaling pathway may play roles in ZNC-mediated treatment of AD. Many previous studies have shown that TCR mediates antigen-specific T cell responses, and TCRs are protein complexes formed by six different polypeptides in the TCR signaling pathway [43, 44]. In people with AD, T cells become more reactive to A*β* [45]. Neuronal loss in AD is due to TNF-mediated necroptosis rather than apoptosis [46]. Accumulating evidence shows an important link between TNF and AD, and the TNF-*α* signaling pathway predominantly mediates inflammatory and proapoptotic signaling pathways [47]. In addition, TNF is involved in systemic inflammation; TNF and TNF receptor type 1 participate in neuroinflammation associated with AD and are also involved in amyloid-*β* formation [48]. MAPKs are serine-threonine kinases in eukaryotes that mediate a wide variety of intracellular processes, including cell proliferation, differentiation, survival, death, and transformation [49]. Furthermore, the MAPK pathway is involved in the pathogenesis of AD through multiple downstream signaling pathways, including the induction of neuronal apoptosis [50]. The above findings indicate that ZNC may ameliorate AD by regulating TCR, TNF, and the MAPK signaling pathway.

Molecular docking between 10 targets with a high degree of PPI and their respective components was evaluated. Then, four key targets with low to high binding affinity according to the measured ΔG kcal/mol, including CASP3, VEGFA, MAPK1, and TNF-*α*, were validated by Western blotting. A recent study revealed that CASP3 is the most important enzyme in apoptosis, and when activated, A*β* can induce neuronal apoptosis [51]. In addition, two other studies showed that CASP3 mediates the cleavage of amyloid precursor protein (APP) to yield neurotoxic peptide fragments, which leads to an increase in production of beta-amyloid [52, 53]. A previous study showed that VEGFA plays an important role in angiogenesis, which has a protective effect in people at high risk of AD [54]. Research suggests that miR-132 may improve the cognition of rats with AD by inhibiting the MAPK signaling pathway [55]. Furthermore, MAPK1 is related to the hyperphosphorylation of Tau protein, which is considered an important factor leading to the neuropathological changes in AD [56]. Another key target is the cytokine TNF-*α*, which is involved in inflammation throughout the body [57]. Furthermore, genetic and epidemiological evidence indicate that an increase in TNF-*α* expression is a risk factor for AD, and exacerbates A*β* and tau pathologies [58]. Therefore, ZNC may act on targets such as CASP3, VEGFA, MAPK1, and TNF-*α* to treat AD.

The results of the validation experiment confirmed that the expression of VEGFA and MAPK1 was significantly upregulated after ZNC administration. That finding suggests that the active components of ZNC could prevent A*β*-induced neuronal injury through the MAPK signaling pathway. In addition, the expression of CASP3 and TNF-*α* was significantly downregulated after ZNC administration. This finding suggests that the active components of ZNC may inhibit apoptosis through the TCR and TNF-*α* signaling pathways.

## 5. Conclusion

In conclusion, the active components of ZNC, such as beta-sitosterol, quercetin, and baicalein may act on multiple targets like CASP3, VEGFA, MAPK1, and TNF-*α* to affect the TCR, TNF, and the MAPK signaling pathway. With the help of network pharmacology and *in vivo* verification experiment, it is useful for us to further understand the influence of ZNC on AD and its potential molecular mechanism. Nevertheless, there are still some limitations in the study. First, the role of key active components should be further validated. Second, data sources mostly rely on specific databases, and frequent updates of the databases are necessary. In future research, our groups will carry out more comprehensive experimental research on ZNC and fully analyze its mechanism of action.

## Figures and Tables

**Table 1:** Targets of above-average node degree ranking.

Name	Degree	Name	Degree	Name	Degree	Name	Degree
AKT1	104	IL1B	75	PTEN	61	ADIPOQ	49
IL6	99	EGFR	75	RELA	60	CRP	48
TNF	95	CAT	71	ICAM1	59	CAV1	48
MAPK3	94	CCL2	71	ERBB2	58	STAT1	48
VEGFA	93	CXCL8	70	CASP8	58	IFNG	47
CASP3	90	CCND1	69	HMOX1	57	NR3C1	47
TP53	88	ESR1	68	SERPINE1	57	CDKN1A	47
JUN	88	PPARG	67	IL4	56	SOD1	45
MAPK8	83	MAPK14	67	AR	54	NFKBIA	45
MAPK1	83	CYCS	66	HIF1A	54	MMP3	44
EGF	80	NOS3	66	APP	53	NOS2	44
FOS	78	IL10	66	SPP1	53	MMP1	44
PTGS2	78	MMP2	65	MPO	51	--	--
MMP9	76	SIRT1	64	IL2	50	--	--
MYC	76	MTOR	63	CASP9	50	--	--

**Table 2:** Results of the molecular docking of four key targets with components.

Target	Component	MOL ID	Binding energy (kcal/mol)
CASP3	Beta-sitosterol	MOL002714	−8.15
CASP3	Naringenin	MOL004328	−7.38
CASP3	Luteolin	MOL000006	−7.34
CASP3	Quercetin	MOL000098	−7.21
CASP3	Baicalein	MOL002714	−7.12
CASP3	Kaempferol	MOL000422	−7.08
TNF-*α*	Quercetin	MOL000098	−7.97
TNF-*α*	Kaempferol	MOL000422	−7.58
TNF-*α*	Luteolin	MOL000006	−7.56
VEGFA	Baicalein	MOL002714	−7.81
VEGFA	Quercetin	MOL000098	−7.56
VEGFA	Diosgenin	MOL000546	−7.39
VEGFA	Luteolin	MOL000006	−7.33
MAPK1	Quercetin	MOL000098	−7.65
MAPK1	Luteolin	MOL000006	−7.44
MAPK1	Naringenin	MOL004328	−7.23

## Data Availability

The data used to support this study are available from the corresponding author upon request.

## Abbreviations

ZNC: Zhinao capsule
AD: Alzheimer's disease
TCMSP: Traditional Chinese Medicine Systems Pharmacology Database
PPI: Protein-protein interaction
GO: Gene ontology
KEGG: Kyoto Encyclopedia of Genes and Genomes
BP: Biological process
CC: Cellular component
MF: Molecular function
CASP3: Caspase-3
TNF-*α*: Tumor necrosis factor-alpha
VEGFA: Vascular endothelial growth factor A
MAPK1: Mitogen-activated protein kinase 1
TCR: T cell receptor
NFT: Neurofibrillary tangle
TCM: Traditional Chinese medicine
TNF: Tumor necrosis factor
AGE: Advanced glycation end products
OB: Oral bioavailability
DL: Drug-likeness
PDB: Protein Data Bank
ANOVA: Analysis of variance
LSD: Least significant difference
A*β*: Amyloid-*β*
PDE2: Phosphodiesterase 2
NLPR3: Nod-like receptor protein 3
APP: Amyloid precursor protein.

## Supplementary Materials

Supplementary Materials Supplementary materials include Supplementary Figure S1, Supplementary Table 1, and Supplementary Table 2. Figure S1: the experimental protocol was approved by the Animal Ethics Committee of Anhui University of Chinese Medicine. Table S1: information on 55 potential components in ZNC. Table S2: results of the molecular docking of 10 targets with components.
