# Supplementary material for: Mechanism of Zhinao Capsule in Treating Alzheimer's Disease Based on Network Pharmacology Analysis and Molecular Docking Validation
Source: J Healthc Eng. 2022 Aug 18;2022:5708769. doi: 10.1155/2022/5708769 (PMC9410932; doi:10.1155/2022/5708769)
Supplement: Supplementary Materials — include Supplementary Figure S1, Supplementary Table 1, and Supplementary Table 2. Figure S1: the experimental protocol was approved by the Animal Ethics Committee of Anhui University of Chinese Medicine. Table S1: information on 55 potential components in ZNC. Table S2: results of the molecular docking of 10 targets with components. [file 5708769.f1.zip › 5708769.f1/Supplementary Figure 1.pdf]

Reply of Experimental Animal Ethics Committee of Anhui University of Chinese Medicine to Effects of Mechanism of Zhinao Capsule in Treating Alzheimer's disease Based on Network Pharmacology Analysis and Molecular Docking Validation

Supervisor of Experimental Animal Ethics Committee of Anhui University of Chinese Medicine,

The experimental animal ethics committee of anhui university of Chinese medicine demonstrated the feasibility of the study “Mechanism of Zhinao Capsule in Treating Alzheimer's disease Based on Network Pharmacology Analysis and Molecular Docking Validation” by Dr. Yanzhen Ma, Hui Jiang, Experimental Center of Clinical Research, The First Affiliated Hospital of Anhui University of Chinese Medicine. The answers are as follows:

On the basis of the introduction of the research plan and information about informed consent, we believe that the subject meets the requirements of animal ethics in the selection of animal species, quantity design, feeding and modeling, therapeutic materials and execution, etc. After the committee’s argument, we agreed on the study of “Mechanism of Zhinao Capsule in Treating Alzheimer's disease Based on Network Pharmacology Analysis and Molecular Docking Validation” by by Dr. Yanzhen Ma, Hui Jiang and to carry out research by Experimental Center of Clinical Research, The First Affiliated Hospital of Anhui University of Chinese Medicine. .

Experimental Animal Ethics Committee of Anhui University of Chinese Medicine

Sept. 13, 2021
