# Supplementary material for: Mechanism of Zhinao Capsule in Treating Alzheimer's Disease Based on Network Pharmacology Analysis and Molecular Docking Validation
Source: J Healthc Eng. 2022 Aug 18;2022:5708769. doi: 10.1155/2022/5708769 (PMC9410932; doi:10.1155/2022/5708769)
Supplement: Supplementary Materials — include Supplementary Figure S1, Supplementary Table 1, and Supplementary Table 2. Figure S1: the experimental protocol was approved by the Animal Ethics Committee of Anhui University of Chinese Medicine. Table S1: information on 55 potential components in ZNC. Table S2: results of the molecular docking of 10 targets with components. [file 5708769.f1.zip › 5708769.f1/Supplementary Table 1 (1).docx]

**Supplementary materials**

**Table S1 Information on 55 potential components in ZNC**

| Components | Mol ID | Molecule Name | OB(%) | DL | Source |
| --- | --- | --- | --- | --- | --- |
| DS1 | MOL001006 | poriferasta-7,22E-dien-3beta-ol | 42.98 | 0.8 | *Codonopsis pilosula* |
| DS2 | MOL002879 | Diop | 43.59 | 0.4 | *Codonopsis pilosula* |
| DS3 | MOL003036 | ZINC03978781 | 43.83 | 0.8 | *Codonopsis pilosula* |
| DS4 | MOL000449 | Stigmasterol | 43.83 | 0.8 | *Codonopsis pilosula* |
| DS5 | MOL003896 | 7-Methoxy-2-methyl isoflavone | 42.56 | 0.2 | *Codonopsis pilosula* |
| DS6 | MOL004355 | Spinasterol | 42.98 | 0.8 | *Codonopsis pilosula* |
| DS7 | MOL005321 | Frutinone A | 65.9 | 0.3 | *Codonopsis pilosula* |
| DS8 | MOL000006 | luteolin | 36.16 | 0.3 | *Codonopsis pilosula* |
| DS9 | MOL006774 | stigmast-7-enol | 37.42 | 0.8 | *Codonopsis pilosula* |
| DS10 | MOL007059 | 3-beta-Hydroxymethyllenetanshiquinone | 32.16 | 0.4 | *Codonopsis pilosula* |
| DS11 | MOL007514 | stigmast-7-enol | 39.67 | 0.2 | *Codonopsis pilosula* |
| DS12 | MOL008400 | glycitein | 50.48 | 0.2 | *Codonopsis pilosula* |
| DS13 | MOL008407 | (8S,9S,10R,13R,14S,17R)-17-[(E,2R,5S)-5-ethyl-6-methylhept-3-en-2-yl]-10,13-dimethyl-1,2,4,7,8,9,11,12,14,15,16,17-dodecahydrocyclopenta[a]phenanthren-3-one | 45.4 | 0.8 | *Codonopsis pilosula* |
| DS14 | MOL008411 | 11-Hydroxyrankinidine | 40 | 0.7 | *Codonopsis pilosula* |
| CX1 | MOL001494 | Mandenol | 42 | 0.2 | *Ligusticum chuanxiong* |
| CX2 | MOL002135 | Myricanone | 40.6 | 0.5 | *Ligusticum chuanxiong* |
| CX3 | MOL002157 | wallichilide | 42.31 | 0.7 | *Ligusticum chuanxiong* |
| HJ1 | MOL001792 | DFV | 32.76 | 0.2 | *Polygonatum sibiricum* |
| HJ2 | MOL002714 | baicalein | 33.52 | 0.2 | *Polygonatum sibiricum* |
| HJ3 | MOL002959 | 3'-Methoxydaidzein | 48.57 | 0.2 | *Polygonatum sibiricum* |
| HJ4 | MOL004941 | (2R)-7-hydroxy-2-(4-hydroxyphenyl)chroman-4-one | 71.12 | 0.2 | *Polygonatum sibiricum* |
| HJ5 | MOL000546 | diosgenin | 80.88 | 0.8 | *Polygonatum sibiricum* |
| HJ6 | MOL006331 | 4',5-Dihydroxyflavone | 48.55 | 0.2 | *Polygonatum sibiricum* |
| HJ7 | MOL009763 | (+)-Syringaresinol-O-beta-D-glucoside | 43.35 | 0.8 | *Polygonatum sibiricum* |
| HQ1 | MOL000211 | Mairin | 55.38 | 0.8 | *Astragali membranaceus* |
| HQ2 | MOL000239 | Jaranol | 50.83 | 0.3 | *Astragali membranaceus* |
| HQ3 | MOL000296 | hederagenin | 36.91 | 0.8 | *Astragali membranaceus* |
| HQ4 | MOL000033 | (3S,8S,9S,10R,13R,14S,17R)-10,13-dimethyl-17-[(2R,5S)-5-propan-2-yloctan-2-yl]-2,3,4,7,8,9,11,12,14,15,16,17-dodecahydro-1H-cyclopenta[a]phenanthren-3-ol | 36.23 | 0.8 | *Astragali membranaceus* |
| HQ5 | MOL000354 | isorhamnetin | 49.6 | 0.3 | *Astragali membranaceus* |
| HQ6 | MOL000371 | 3,9-di-O-methylnissolin | 53.74 | 0.5 | *Astragali membranaceus* |
| HQ7 | MOL000378 | 7-O-methylisomucronulatol | 74.69 | 0.3 | *Astragali membranaceus* |
| HQ8 | MOL000379 | 9,10-dimethoxypterocarpan-3-O-β-D-glucoside | 36.74 | 0.9 | *Astragali membranaceus* |
| HQ9 | MOL000380 | (6aR,11aR)-9,10-dimethoxy-6a,11a-dihydro-6H-benzofurano[3,2-c]chromen-3-ol | 64.26 | 0.4 | *Astragali membranaceus* |
| HQ10 | MOL000387 | Bifendate | 31.1 | 0.7 | *Astragali membranaceus* |
| HQ11 | MOL000392 | formononetin | 69.67 | 0.2 | *Astragali membranaceus* |
| HQ12 | MOL000417 | Calycosin | 47.75 | 0.2 | *Astragali membranaceus* |
| HQ13 | MOL000439 | isomucronulatol-7,2'-di-O-glucosiole | 49.28 | 0.6 | *Astragali membranaceus* |
| HQ14 | MOL000442 | 1,7-Dihydroxy-3,9-dimethoxy pterocarpene | 39.05 | 0.5 | *Astragali membranaceus* |
| RCR1 | MOL005320 | arachidonate | 45.57 | 0.2 | *Cistanche deserticola* |
| RCR2 | MOL005384 | suchilactone | 57.52 | 0.6 | *Cistanche deserticola* |
| RCR3 | MOL007563 | Yangambin | 57.53 | 0.8 | *Cistanche deserticola* |
| RCR4 | MOL008871 | Marckine | 37.05 | 0.7 | *Cistanche deserticola* |
| SCP1 | MOL003542 | 8-Isopentenyl-kaempferol | 38.04 | 0.4 | *Acorus tatarinowii* |
| SCP2 | MOL003576 | (1R,3aS,4R,6aS)-1,4-bis(3,4-dimethoxyphenyl)-1,3,3a,4,6,6a-hexahydrofuro[4,3-c]furan | 52.35 | 0.6 | *Acorus tatarinowii* |
| SCP3 | MOL003578 | Cycloartenol | 38.69 | 0.8 | *Acorus tatarinowii* |
| YJ1 | MOL004328 | naringenin | 59.29 | 0.2 | *Curcuma wenyujin* |
| A1 | MOL000358 | beta-sitosterol | 36.91 | 0.8 | *Polygonatum sibiricum*  *Cistanche deserticola*  *Curcuma wenyujin* |
| B1 | MOL000359 | sitosterol | 36.91 | 0.8 | *Ligusticum chuanxiong*  *Polygonatum sibiricum*  *Curcuma wenyujin* |
| C1 | MOL000098 | quercetin | 46.43 | 0.3 | *Cistanche deserticola*  *Astragali membranaceus* |
| D1 | MOL002140 | Perlolyrine | 65.95 | 0.3 | *Codonopsis pilosula*  *Ligusticum chuanxiong* |
| E1 | MOL000422 | kaempferol | 41.88 | 0.2 | *Astragali membranaceus*  *Acorus tatarinowii* |
| F1 | MOL000433 | FA | 68.96 | 0.7 | *Astragali membranaceus*  *Ligusticum chuanxiong* |
| DL1 | MOL000061 | proline | -- | -- | *Pheretima aspergillum* |
| DL2 | MOL000067 | valine | -- | -- | *Pheretima aspergillum* |
| DL3 | MOL000054 | arginine | -- | -- | *Pheretima aspergillum* |
