# Supplementary material for: Mechanism of Zhinao Capsule in Treating Alzheimer's Disease Based on Network Pharmacology Analysis and Molecular Docking Validation
Source: J Healthc Eng. 2022 Aug 18;2022:5708769. doi: 10.1155/2022/5708769 (PMC9410932; doi:10.1155/2022/5708769)
Supplement: Supplementary Materials — include Supplementary Figure S1, Supplementary Table 1, and Supplementary Table 2. Figure S1: the experimental protocol was approved by the Animal Ethics Committee of Anhui University of Chinese Medicine. Table S1: information on 55 potential components in ZNC. Table S2: results of the molecular docking of 10 targets with components. [file 5708769.f1.zip › 5708769.f1/Supplementary Table 2 (1).docx]

**Supplementary materials**

**Table S2 Results of the molecular docking of 10 targets with components**

| **Component** | **MOL ID** | **Binding energy(kcal/mol)** | | | | | | | | | |
| --- | --- | --- | --- | --- | --- | --- | --- | --- | --- | --- | --- |
|  |  | **AKT1** | **IL6** | **TNF-α** | **MAPK3** | **VEGFA** | **CASP3** | **TP53** | **JUN** | **MAPK8** | **MAPK1** |
| luteolin  baicalein  diosgenin  kaempferol  quercetin  naringenin  beta-sitosterol  formononetin | MOL000006  MOL002714  MOL000546  MOL000422  MOL000098  MOL004328  MOL000358  MOL000392 | -7.26  -7.24  -6.49  -6.92  -6.99  -6.75  NA  NA | -7.12  NA  NA  NA  -6.92  NA  NA  NA | -7.56  NA  NA  -7.58  -7.97  NA  NA  NA | NA  NA  NA  NA  NA  -6.97  NA  NA | -7.33  -7.81  -7.39  NA  -7.56  NA  NA  NA | -7.34  -7.12  NA  -7.08  -7.21  -7.38  -8.15  NA | -7.04  -6.61  -6.61  NA  -7.16  NA  NA  NA | -6.34  NA  NA  -6.47  -6.61  NA  -6.15  -6.44 | NA  -7.12  NA  NA  NA  NA  NA  NA | -7.44  NA  NA  NA  -7.65  -7.23  NA  NA |
|  |  |  |  |  |  |  |  |  |  |  |  |
